# Screening of commonly prescribed drugs for effects on the CAT1-mediated transport of l-arginine and arginine derivatives

**DOI:** 10.1007/s00726-022-03156-2

**Published:** 2022-04-04

**Authors:** Sofna Banjarnahor, Jörg König, Renke Maas

**Affiliations:** 1 Institute of Experimental and Clinical Pharmacology and Toxicology, Friedrich-Alexander-Universität Erlangen-Nürnberg, 91054 Erlangen, Germany; 2 Research Centre for Chemistry, The National Research and Innovation Agency (BRIN), Kawasan PUSPIPTEK Serpong, Tangerang Selatan, Banten 15314 Indonesia

**Keywords:** CAT1, l-Arginine, l-Homoarginine, ADMA, Inhibition

## Abstract

The cationic amino acid transporter 1 (CAT1/*SLC7A1*) plays a key role in the cellular uptake or export of l-arginine and some of its derivatives. This study investigated the effect of 113 chemically diverse and commonly used drugs (at 20 and 200 µM) on the CAT1-mediated cellular uptake of l-arginine, l-homoarginine, and asymmetric dimethylarginine (ADMA). Twenty-three (20%) of the tested substances showed weak inhibitory or stimulatory effects, but only verapamil showed consistent inhibitory effects on CAT1-mediated transport of all tested substrates.

**Supplementary Information:**

The online version contains supplementary material available at 10.1007/s00726-022-03156-2.

## Introduction

The cationic amino acid transporter 1 (CAT1/*SLC7A1*) is a member of the solute carrier protein (SLC) superfamily, expressed in most human tissues (Closs et al. 2004) and is considered a crucial, if not the primary uptake transporter of l-arginine in many tissues (Closs 1996). l-arginine is a key physiological substrate of endothelial nitric oxide synthase (eNOS) (Siasos et al. 2007). Thus, the alteration of its availability may significantly affect l-arginine-dependent NO signaling in health and disease. However, it has been observed that some l-arginine derivatives such as l-homoarginine are substrates for mouse Cat1 (Kim et al. 1991) and human CAT1 (Chafai et al. 2017). Asymmetric dimethylarginine (ADMA) is also a substrate of human CAT1 (Strobel et al. 2012).

Among the l-arginine-related CAT1 substrates (Banjarnahor et al. 2020), ADMA has gained interest as a risk marker (Vallance et al. 1992; Böger et al. 2009). ADMA may act as a competitive inhibitor of nitric oxide synthase (NOS), and elevated plasma concentrations of ADMA are independently associated with increased mortality (Schlesinger et al. 2016). In contrast, emerging evidence indicates that l-homoarginine is a protective marker for cardiovascular and total mortality (Atzler et al. 2014; März et al. 2010). Despite the vital role of l-arginine itself and the emerging importance of l-homoarginine and ADMA, published data regarding potential off-target effects of commonly used drugs on CAT1-mediated uptake of l-arginine, l-homoarginine, and ADMA are rather limited.

In contrast to classic drug transporters (e.g., OATP1B1, OCT2, or P-gp), where transport inhibition leads to potential drug–drug interactions and may cause changes in drug pharmacokinetics (Müller and Fromm 2011), inhibition of amino acid transporters is expected to alter the substrate homeostasis. The physiological effects of l-arginine, ADMA, and l-homoarginine, as well as the homeostasis of their plasma concentrations, depend on their transport protein-mediated distribution between net generating and net metabolizing tissues (Banjarnahor et al. 2020). While the effects of various pathophysiological conditions on the expression and function of CAT1 have been explored (Lu et al. 2013; Tun and Kang 2017; Toral et al. 2018; Grupper et al. 2013; Bentur et al. 2015), the effects of commonly used drugs on CAT1-mediated uptake are largely unknown. Therefore, this study aimed to evaluate whether CAT1 transport activity may be altered by marketed drugs.

## Materials and methods

### Chemicals

[^3^H]‐l-arginine (43 Ci/mmol) was obtained from American Radiolabeled Chemicals, Inc (St. Louis, MO, USA), **[**^3^H]-l-homoarginine (6 Ci/mmol) was obtained from ViTrax Co (CA, USA), [^3^H]‐ADMA (25 Ci/mmol) was purchased from BIOTREND Chemikalien GmbH (Cologne, Germany). Unlabeled substrates, library drugs, reagents, and solvents were purchased from commercial suppliers and used as received unless otherwise noted. Each of the pharmaceutical compounds was of analytical grade. The drugs used were selected based on a representative set of small molecule drugs previously shown suitable to investigate drug effects on organic cationic transporter 2 (OCT2)-mediated transport (Hacker et al. 2015). The drug concentrations of 20 μM and 200 μM were chosen for screening purposes with a focus on the ability to detect possible effects and to avoid overlooking an interaction (Parvez et al. 2016; Hacker et al. 2015). In addition, the respective concentrations were selected, because we deliberately preferred sensitivity in detection of effects at the price of possible overestimation rather than to miss effects. Our focus was on screening effects seen at a high concentration which would elicit a verification at lower concentration. Two inhibitor concentrations also permitted a first indication of a dose–response relation. Any significant finding in the 20 µM/200 µM screening could then be confirmed by more detailed inhibition experiments that also included much lower drug concentrations. Therefore, the concentrations exceeded the expected therapeutic plasma concentrations of the drugs tested. Additionally, the 20 and 200 μM were chosen to allow the identification/exclusion of artifacts caused by quenching and toxicity. The selection of drugs was based on the diversity of substances, clinical relevance, and commercial availability.

### Cell lines

Stably transfected HEK-CAT1 and HEK-VC cells were already established and characterized previously (Strobel et al. 2012). All cell culture media supplements were purchased from Invitrogen GmbH (Karlsruhe, Germany).

### Transport and inhibition studies in HEK-CAT1 Cells

**Fig. 1:**
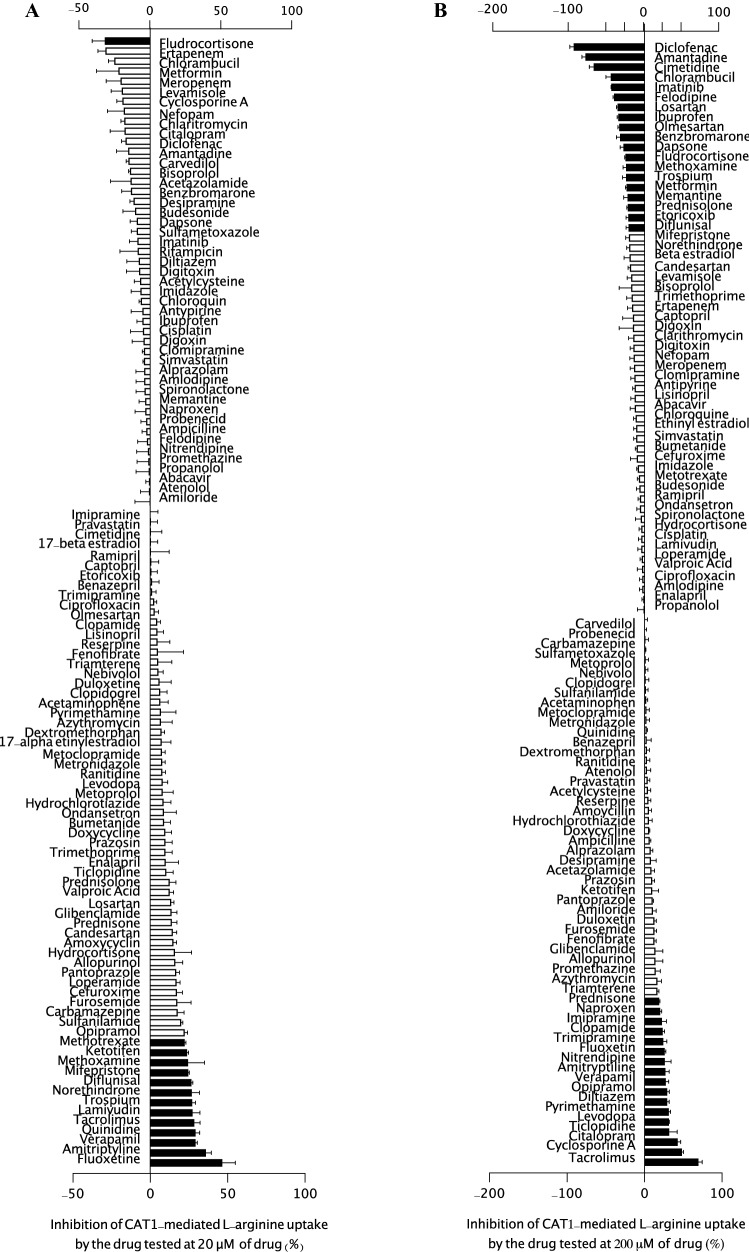
Inhibition of CAT1-mediated uptake of l-arginine (100 μM) in a screen of 113 commonly used drugs at **A** 20 μM and **B** 200 μM (excluding rifampicin). Each bar represents the effect of one compound on CAT1-mediated l-arginine (100 μM) uptake as percent inhibition. Data were obtained from at least two independent experiments performed in triplicate (*n* = 6–9) and each test data from the independent experiment were normalized to the respective control data to get the value of percent inhibition. Negative inhibition values indicate stimulated CAT1-mediated uptake. Data were analyzed using one-way ANOVA and Dunnett’s multiple comparison test with *p* < 0.05. Bars showing a statistically significant inhibition or transport stimulation are shaded in black

**Fig. 2:**
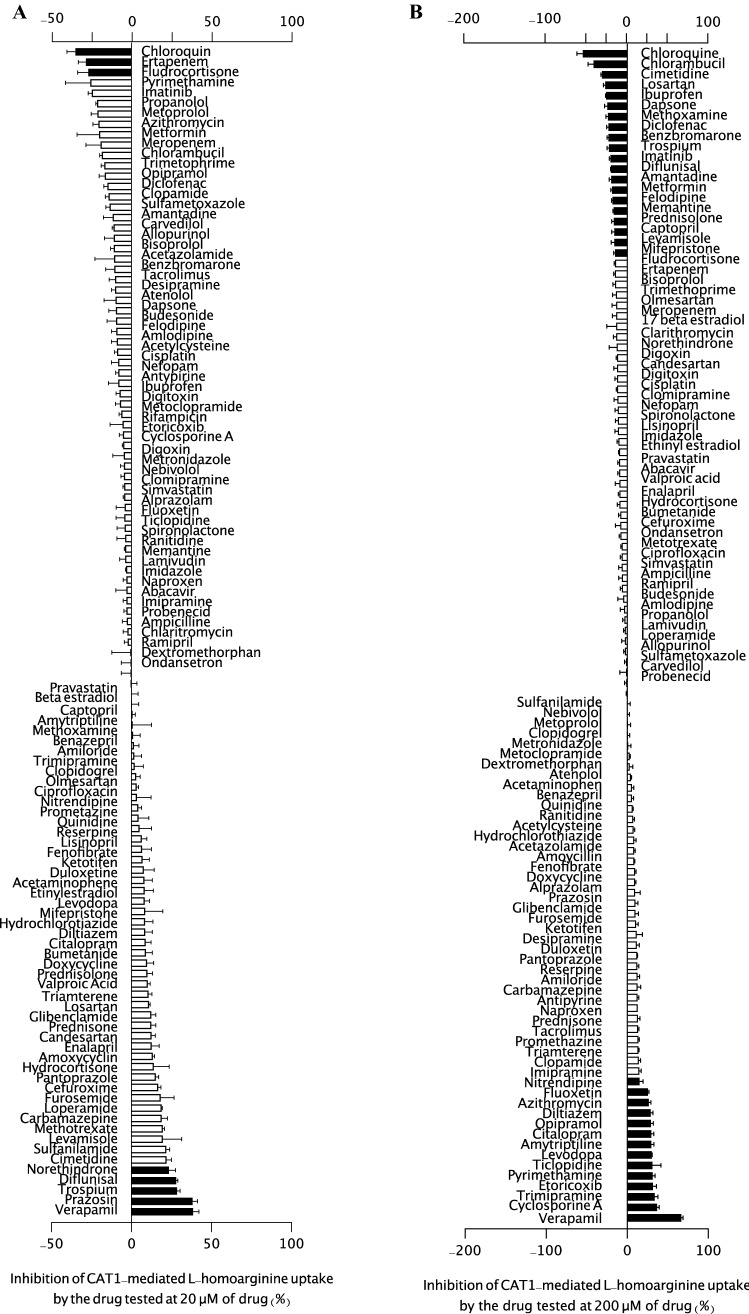
Inhibition of CAT1-mediated uptake of L-homoarginine (1 μM) in a screen of 113 commonly used drugs at **A** 20 μM and **B** 200 μM (excluding rifampicin). Each bar represents the effect of one compound on CAT1-mediated l-homoarginine (1 μM) uptake as percent inhibition. Data were obtained from at least two independent experiments performed in triplicate (*n* = 6–9) and each test data from the independent experiment were normalized to the respective control data to get the value of percent inhibition. Negative inhibition values indicate stimulated CAT1-mediated uptake. Data were analyzed using one-way ANOVA and Dunnett’s multiple comparison test with *p* < 0.05. Bars showing a statistically significant inhibition or transport stimulation are shaded in black

**Fig. 3:**
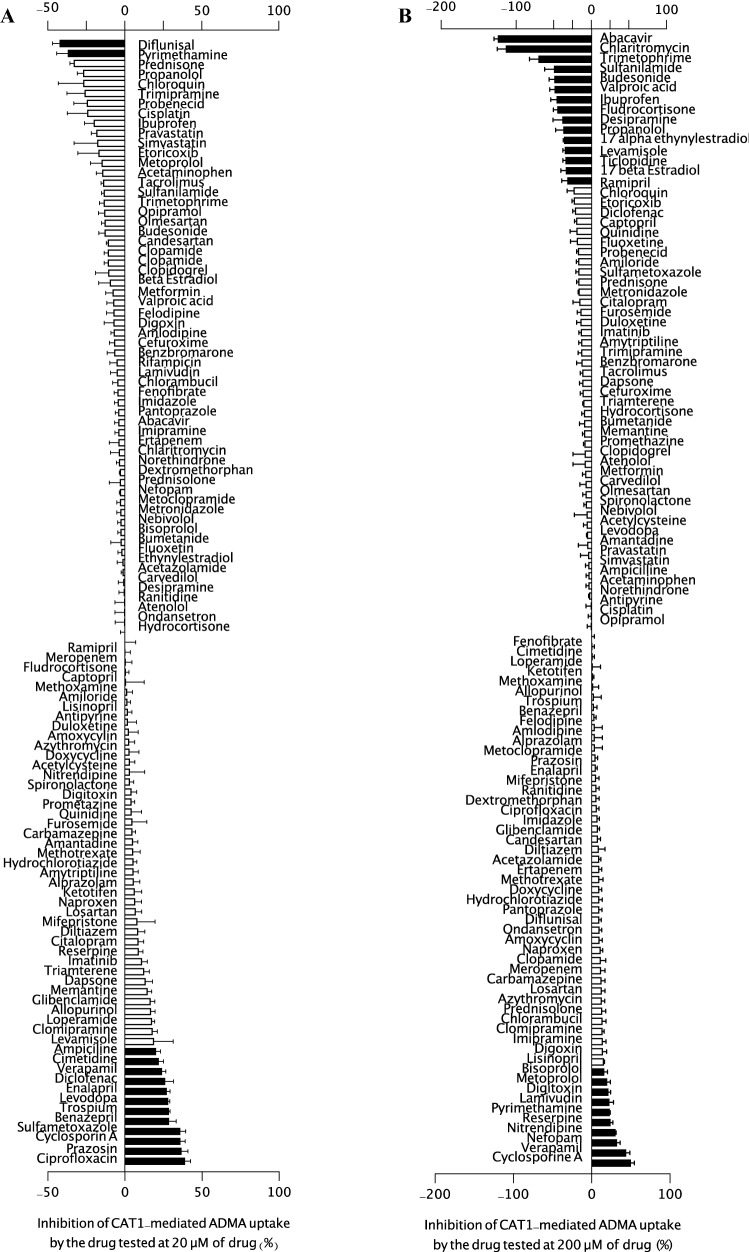
Inhibition of CAT1-mediated uptake of ADMA (1 μM) in a screen of 113 commonly used drugs at **A** 20 μM and **B** 200 μM (excluding rifampicin). Each bar represents the effect of one compound on CAT1-mediated ADMA (1 μM) uptake as percent inhibition. Data were obtained from at least two independent experiments performed in triplicate (*n* = 6–9) and each test data from the independent experiment were normalized to the respective control data to get the value of percent inhibition. Negative inhibition values indicate stimulated CAT1-mediated uptake. Data were analyzed using one-way ANOVA and Dunnett’s multiple comparison test with *p* < 0.05. Bars showing a statistically significant inhibition or transport stimulation are shaded in black

Uptake and inhibition assays using HEK-CAT1 and HEK-VC cells and l-arginine, l-homoarginine, and ADMA as substrates were performed using the protocol described (Strobel et al. 2012; Chafai et al. 2017). Briefly, after incubation, the supernatant was replaced with transport buffer containing [^3^H]- l-arginine for 5 min and 2.5 min for medium containing [^3^H]-l-homoarginine or [^3^H]-ADMA with or without the test compound. The reference drugs were screened at concentrations of 20 µM (Figs. 1A, 2A, and 3A) and 200 µM (Figs. 1B, 2B, and 3B). The substrate accumulation inside the cells was estimated using a liquid scintillation counter (Tri-Carb^®^ 2800 TR Liquid Scintillation Analyzer, Perkin Elmer LAS GmbH). To confirm the experimental setup, similar experiments in the presence of *N*-ethylmaleimide (NEM), a CAT-specific inhibitor (1000 µM) (Deves et al. 1993), were performed. The net uptake was determined by subtracting the total amount taken up by control cells from the amount taken up by CAT1-expressing cells, which was expressed as a percentage of the amount taken up by the control. To determine the inhibitory effects of the drugs on CAT1-mediated transport of l-arginine, l-homoarginine, or ADMA, the control was measured in the absence of the potential inhibitor. The value for the percent inhibition was calculated as: Inhibition (%) = 100 – (*V*/*V*_0_^*^ 100), where *V* and *V*_0_ are the net uptake rates (uptake into HEK-CAT1 cells—uptake into HEK-VC cells) with and without test drugs, respectively. Data are presented as the percent inhibition of the CAT1-mediated uptake of l-arginine, l-homoarginine, or ADMA.

### Statistical analysis

A one-way ANOVA followed by Dunnett’s test was used to determine the statistical significance differences in CAT1-mediated l-arginine, l-homoarginine, or ADMA uptake between control and test drug. Data were presented as mean ± standard error of the mean. Graphs and statistical analysis were conducted using GraphPad Prism^®^ 8.0 software (GraphPad Software Inc.).

## Results

A list of all tested substances together with their ATC code, CAS number, and their effect on CAT1-mediated transport is summarized in Table S1 [Table supplement S1]. As shown in Fig. 1A, the transport activity of CAT1-mediated l-arginine uptake was moderately but significantly inhibited by nine drugs (diflunisal, norethindrone, trospium, lamivudine, tacrolimus, quinidine, verapamil, amitriptyline, and fluoxetine) at 20 µM (25–50% inhibition) and only fludrocortisone stimulated the transport by at least 30%. In contrast, the remaining drugs displayed biologically negligible inhibitory effects on CAT1-mediated l-arginine uptake. The same drugs were also tested against the transport of l-homoarginine (1 µM). Verapamil, prazosin, trospium, diflunisal, and norethindrone used at 20 μM, were the only drugs that significantly inhibited CAT1-mediated transport of l-homoarginine (Fig. 2A); the percentage of inhibition (38.6%, 38.2%, 28.6%, 27.9%, and 23.5%, respectively) was, however, less than 50%. Furthermore, only ten drugs (verapamil, diclofenac, enalapril, levodopa, trospium, benazepril, sulfamethoxazole, cyclosporin A, prazosin, and ciprofloxacin) significantly inhibited ADMA uptake (1 µM) by at least 25–50% (Fig. 3A). Comparing inhibitory effects at 20 µM and 200 µM, no consistent or dose-dependent effects could be observed for most drugs. Only verapamil showed a consistent inhibitory effect across the three substrates. In subsequent inhibition studies, verapamil inhibited CAT1-mediated uptake of l-arginine, l-homoarginine, and ADMA with IC_50_ values of 85.3, 58.1, and 113 µM, respectively [Figure supplement S3]. Lineweaver–Burk plots of l-arginine uptake in the presence of different concentrations of the respective inhibitors showed a competitive inhibition pattern for l-homoarginine and ADMA. *N*-ethylmaleimide (NEM) showed a non-competitive pattern and verapamil an uncompetitive pattern [Figure supplement S4].

## Discussion

The key findings of our studies are that several commonly prescribed drugs at concentrations above those expected as plasma concentrations in their clinical use significantly but not completely inhibit CAT1-mediated cellular uptake of l-arginine, l-homoarginine, and ADMA. Previous studies have shown that CAT1 preferentially accepts cationic amino acids such as l-arginine (Closs et al. 2006) and its derivatives l-homoarginine (Chafai et al. 2017) and ADMA (Strobel et al. 2012) as substrates. So far, the only known inhibitor for CAT1-mediated transport is NEM which acts selectively on the cysteine residues of the transporter (Deves et al. 1993). Possible effects of pharmacotherapies on plasma concentrations of CAT1 substrates, namely l-arginine, l-homoarginine (Maas et al. 2020), and ADMA (Maas 2005), have been reported. Nevertheless, the underlying mechanisms, including effects on CAT1-mediated transport, largely remained to be elucidated.

We found that verapamil inhibits CAT1-mediated transport in an uncompetitive manner which is most likely related to its known effect on the membrane potential. Verapamil alters potential distribution across plasma membrane (Pohl et al. 1998) possibly related to alteration of cell membranes by perturbation of polar heads and acyl chain regions of phospholipid bilayers (Suwalsky et al. 2010). Furthermore, it was shown that conformational changes which involve the translocation of charges or dipoles across the membrane could be strongly affected by the dipole potential (Qin et al. 1995). Additionally, Shi and Tien (Shi and Tien 1986) have shown that the verapamil concentration required to generate the detectable fluidizing effect is around 10 μM (which is exceeded by the concentration used in our experiment). CAT1 as transport system is electrogenic in nature and l-arginine uptake is modified when the plasma membrane potential is depolarized (Zharikov et al. 1997) or hyperpolarized (Bogle et al. 1991). Therefore, whether verapamil indeed influences CAT1 transport activity by modifying plasma membrane potential warrants further research. The IC_50_ value of verapamil for CAT1-mediated l-arginine, l-homoarginine, and ADMA uptake (85.3, 58.1, and 113 µM, respectively) is orders of magnitude higher than the maximum plasma concentrations of free (non-protein bound) verapamil (∼ 0.05 µM) (Regenthal et al. 1999), suggesting that verapamil has no potential to cause significant clinical transport interactions, according to the United States Food and Drug Administration (FDA) guidelines (FDA 2020).

Regarding the current study, it is also of interest that the drugs, fenofibrate and allopurinol, assessed in this study, were previously associated with higher and lower plasma concentrations of l-arginine, respectively (Maas et al. 2020). It was also shown that fenofibrate was independently associated with higher plasma concentrations of l-homoarginine (Maas et al. 2020). In the case of ADMA, drugs such as angiotensin-converting enzyme (ACE) inhibitors, angiotensin receptor blockers (ARBs), hypoglycemic agents, and hormone-replacement therapy were observed to be associated with reduced ADMA plasma levels (Maas 2005; Trocha et al. 2010). Our results indicate that in either case, effects on CAT1-mediated transport are an unlikely explanation.

As we aimed to identify drug effects relevant to patients, we studied the substrates in concentrations that are plausible in vivo (i.e., 100 µM for l-arginine and 1 µM for l-homoarginine and ADMA). The observed drug-CAT1 inhibition, however, occurred at concentrations that are much higher than those commonly seen as plasma concentrations in the clinical use of these drugs, which makes any clinically relevant contribution of these drugs to CAT1 transport-related metabolic aberrations unlikely. Therefore, for some drugs, we may have missed possibly more profound inhibitory effects at concentrations exceeding those seen with normal therapeutic doses of the drugs. The example of verapamil indicates that we might have identified more, albeit weak, inhibitors using much higher concentrations. So far, there are insufficient data for computational analysis and in silico modeling of CAT1 inhibitors available to deduce or model the precise mechanism(s) of inhibition; only the crystal structure of a close homolog of the mammalian CATs is available (Jungnickel et al. 2018).

Screening was adjusted for testing multiple inhibitors, this may have limited our ability to identify more subtle effects as statistically significant, i.e., it may have increased the risk for false negatives. Given the high inhibitor concentrations used in screening, it is unlikely though that effects that still would be relevant at lower concentrations would have been missed. With regard false positive results despite adjustments for multiple testing, these would have been identified in the more detailed assays performed for drugs with significant results in the screening. This is the reason why positive results are then assessed in more detailed experiments. Finally, the aim of the study was to investigate the effects of individual drugs on the CAT1-mediated transport of individual amino acids at their physiological concentrations. By convention other potential competitive compounds are not included into these screening assays in the assay buffer. Assaying more complex compound combinations mimicking plasma was beyond the scope of this screening study. Overall, our explorative study was able to identify verapamil as potential inhibitor of CAT1, which may be of use in experimental settings.

## Supplementary Information

Below is the link to the electronic supplementary material.

Supplementary file (DOCX 481 KB)
