# Supplementary material for: Screening of commonly prescribed drugs for effects on the CAT1-mediated transport of l-arginine and arginine derivatives
Source: Amino Acids. 2022 Apr 4;54(7):1101–8. doi: 10.1007/s00726-022-03156-2 (PMC9217908; doi:10.1007/s00726-022-03156-2)
Supplement: Supplementary file 1 — Supplementary file (DOCX 481 KB) [file 726_2022_3156_MOESM1_ESM.docx]

**Screening of commonly prescribed drugs for effects on the CAT1-mediated transport of L-arginine and arginine derivatives**

Sofna Banjarnahor ^1,2^ . Jörg König ^1^ . and Renke Maas ^1^

^1^ Institute of Experimental and Clinical Pharmacology and Toxicology, Friedrich-Alexander-Universität Erlangen-Nürnberg, 91054 Erlangen, Germany

^2^ Research Centre for Chemistry – The National Research and Innovation Agency (BRIN), Kawasan PUSPIPTEK Serpong, 15314 Tangerang Selatan, Banten, Indonesia

Correspondence: [Renke.Maas@fau.de](mailto:Renke.Maas@fau.de)

| A |  |
| --- | --- |
| B |  |
| C |  |

Figure S1. Time-dependent uptake of L-arginine (A, 100 μM), L-homoarginine (B, 1 μM), and ADMA (C, 1 μM) into CAT1-expressing HEK-cells and corresponding vector controls. Data are presented as the mean ± SEM (two experiments each on two separate days, n = 6). ***P < 0.0001, **P < 0.001, *P < 0.01 (two-tailed t test).

| A |  |
| --- | --- |
| B |  |
| C |  |

Figure S2. Uptake of L-arginine (A, 100 μM), L-homoarginine (B, 1 μM), and ADMA (C, 1 μM) into CAT1-expressing HEK-cells and corresponding vector controls after five minutes for L-arginine and 2.5 minutes for L-homoarginine and ADMA. Data are presented as the mean ± SEM (two experiments each on two separate days, n = 6). ***P < 0.0001 (two-tailed t test).

| A  | B  |
| --- | --- |
| C   | |

Figure S3. Concentration-dependent inhibition of CAT1-mediated (A) L-arginine (100 μM), (B) L-homoarginine (1 μM), and (C) ADMA (1 μM) uptake by verapamil. The calculated uptake values were used to determine IC_50_ values. Data are expressed as means ± SEM. Experiments were performed on two separate days with n = 3 (n = 6). *P < 0.05; ***P < 0.0001; one-way ANOVA Dunnett`s multiple comparison test.

Figure S4**.** Lineweaver-Burk plots of CAT1-mediated L-arginine uptake showing competitive inhibition by its derivatives (A) L-homoarginine and (B) ADMA; (C) non-competitive inhibition by N-ethylmaleimide (NEM); (D) uncompetitive inhibition by verapamil. L-homoarginine (0, 300, 1000, and 3000 μM), ADMA (0, 300, 1000, and 3000 μM), NEM (0, 500, and 1000 μM), Verapamil (0, 20, and 200 μM), and L-arginine (100, 300, 1000, and 3000 μM) concentrations were used. Each point from two to three independent experiments in triplicate.

Table S1. List of commonly prescribed drugs which were included in the screening library with their Anatomical Therapeutic Chemical (ATC) codes and Chemical Abstracts Service (CAS) numbers and experimentally determined inhibition of CAT1 by these compounds. Bold letters represent significant inhibition at the respective concentration. Negative values for “% inhibition” indicate an increase in transport activity. (*) data for rifampicin are only shown for matters of completeness because at a high concentration of 200 µM it causes a technical artifact due to color quenching of scintillation signals, rather than an actual effect on transport.

| No | Drug | ATC code | CAS number | % Inhibition of CAT1-mediated  L-arginine uptake | | | % Inhibition of CAT1-mediated  L-homoarginine uptake | | % Inhibition of CAT1-mediated ADMA uptake | |
| --- | --- | --- | --- | --- | --- | --- | --- | --- | --- | --- |
|  |  |  |  | at 20 μM  (mean ± SEM) | at 200 μM  (mean ± SEM) | at 20 μM  (mean ± SEM) | | at 200 μM  (mean ± SEM) | at 20 μM  (mean ± SEM) | at 200 μM  (mean ± SEM) |
| 1 | Abacavir | J05AF06 | 188062-50-2 | -0.9 ± 1.81 | -12.4 ± 6 | -3.1 ± 2 | | -9.8 ± 1.7 | -4.3 ± 2.2 | -78.1 ± 1.9 |
| 2 | Acetaminophen | N02BE01 | 103-90-2 | 6.6 ± 4.9 | 2.4 ± 1.6 | 8.1 ± 4.7 | | 4.4 ± 0.7 | -14.7 ± 3.8 | -3.9 ± 3.4 |
| 3 | Acetylcysteine | R05CB01 | 616-91-1 | -6.6 ± 3.7 | 4.6 ± 3.3 | -9 ± 1.5 | | 7.5 ± 1.3 | 3.2 ± 3.2 | -6.1 ± 4.3 |
| 4 | Acetazolamide | S01EC01 | 59-66-5 | -12.8 ± 12.9 | 8.9 ± 3.9 | -10.9 ± 11.9 | | 8.8 ± 2.1 | -1.5 ± 0.9 | 9.7 ± 1.9 |
| **5** | **Allopurinol** | M04AA01 | 315-30-0 | 5 ± 16.4 | 14.1 ± 9.6 | **46.5 ± 5.8** | | -2.6 ± 1.6 | 16.9 ± 2.6 | 2 ± 7 |
| 6 | Alprazolam | N05BA12 | 28981-97-7 | -3.9 ± 5.3 | 8 ± 2.7 | -4.3 ± 5.2 | | 9.6 ± 1 | 5.8 ± 3.8 | 4.1 ± 9.5 |
| 7 | Amantadine | N04BB01 | 665-66-7 | -14.2 ± 7.4 | -76 ± 4.5 | 11.9 ± 5.6 | | -19.7 ± 2.6 | 5.3 ± 3 | -5.7 ± 11.1 |
| 8 | Amiloride | C03DB01 | 2016-88-8 | -0.1 ± 9.7 | 10.9 ± 3.9 | 1.5 ± 3.2 | | 12.6 ± 2.6 | 1.5 ± 3.2 | -17.1 ± 2.3 |
| **9** | **Amitriptyline** | N06AA09 | 549-18-8 | **35.9 ± 3.6** | **26.9 ± 4.7** | 0.6 ± 1.7 | | **29.7 ± 3.2** | 5.6 ± 2.9 | -13.4 ± 3 |
| 10 | Amlodipine | C08CA01 | 88150-42-9 | -3.9 ± 5.2 | -2.6 ± 3.9 | -9.3 ± 3.3 | | -3.9 ± 4.6 | -7.3 ± 1.7 | 4 ± 9.5 |
| 11 | Amoxicillin | J01CA04 | 26787-78-0 | 14.9 ± 2 | 5.9 ± 2.9 | 13.3 ± 1 | | 8.8 ± 1.1 | 2.7 ± 5.9 | 10.2 ± 3.2 |
| **12** | **Ampicillin** | J01CA01 | 69-52-3 | -2.5 ± 2.7 | 6.7 ± 1.4 | -2.5 ± 2.7 | | -6.6 ± 3.6 | **20.2 ± 2.8** | -4.1 ± 2.8 |
| 13 | Antipyrine | N02BB01 | 60-80-0 | -5.1 ± 7.2 | -12.7 ± 2.5 | -8.2 ± 6 | | 12.8 ± 1.4 | 1.9 ± 2.5 | -1.2 ± 6.1 |
| 14 | Atenolol | C07AB03 | 29122-68-7 | -0.8 ± 5.3 | 3 ± 4.7 | -10 ± 7.1 | | 3.1 ± 3.7 | -0.5 ± 5.9 | -8.9 ± 14.8 |
| **15** | **Azithromycin** | J01FA10 | 83905-01-5 | 7 ± 7.2 | 16.7 ± 5.2 | 20.8 ± 3.3 | | **26.8 ± 2.3** | 2.8 ± 3.4 | 12.9 ± 3.7 |
| **16** | **Benazepril** | C09AA07 | 86541-74-4 | 1.1 ± 4.5 | 2.7 ± 6 | 1.1 ± 4.5 | | 5.8 ± 2.4 | **28.7 ± 4.6** | 3.1 ± 3.8 |
| 17 | Beta estradiol | G03CA03 | 50-28-2 | 0.3 ± 4.5 | -19.1 ± 7.2 | -0.1 ± 4.2 | | -13.7 ± 11.3 | -9.8 ± 7.2 | -32.9 ± 6.4 |
| 18 | Benzbromarone | M04AB03 | 3562-84-3 | -12.4 ± 6.1 | -31.3 ± 4.9 | -10.8 ± 5.3 | | -23.2 ± 1.6 | -6.9 ± 4.6 | -13.2 ± 5.9 |
| **19** | **Bisoprolol** | C07AB07 | 66722-44-9 | -13.1 ± 0.7 | -17.2 ± 15.5 | -11.1 ± 1.9 | | -14.9 ± 2.5 | -2.8 ± 1.6 | **16.2 ± 4.2** |
| 20 | Budesonide | R01AD05 | 51333-22-3 | -9.7 ± 7.8 | -6.4 ± 3.9 | -9.7 ± 5.5 | | -4.9 ± 6.5 | -13.2 ± 3.7 | -47.7 ± 6.5 |
| 21 | Bumetanide | C03CA02 | 28395-03-1 | 9 ± 4.0 | -10.1 ± 1.7 | 8.9 ± 4.1 | | -8.6 ± 1.9 | -2.8 ± 6.2 | -9.7 ± 5.6 |
| 22 | Candesartan | C09CA06 | 139418-59-7 | 14.5 ± 2.4 | -18.8 ± 2.4 | 12.6 ± 2.2 | | -12.6 ± 3.8 | -11.1 ± 0.9 | 8.1 ± 3.1 |
| 23 | Captopril | C09AA01 | 62571-86-2 | 0.6 ± 4.7 | -15.1 ± 13.1 | 0.1 ± 4.5 | | -15.9 ± 3 | 0.5 ± 2.1 | -19.6 ± 2.2 |
| 24 | Carbamazepine | N03AF01 | 298-46-4 | 17.8 ± 4.1 | 0.7 ± 4.3 | 18.9 ± 3.5 | | 12.7 ± 3.9 | 4.8 ± 2.1 | 12.8 ± 4.2 |
| 25 | Carvedilol | C07AG02 | 72956-09-3 | -14.1 ± 1.5 | 0.2 ± 3.5 | -11.2 ± 0.9 | | -1.4 ± 7.7 | -1.1 ± 3.1 | -7.8 ± 7.1 |
| 26 | Cefuroxime | J01DC02 | 55268-75-2 | 17.3 ± 3.5 | -9.8 ± 8.4 | 16.8 ± 1.6 | | -8.5 ± 5.9 | -7.1 ± 2.9 | -11.5 ± 2.8 |
| 27 | Chlorambucil | L01AA02 | 305-03-3 | -23.3 ± 3.8 | -43.3 ± 6.5 | -18.6 ± 1.4 | | -41.6 ± 6.4 | -5.1 ± 2.8 | 13.9 ± 4.4 |
| 28 | Chloroquine | P01BA01 | 50-36-5 | -6.1 ± 1.1 | -11.4 ± 2.6 | -35.1 ± 5.2 | | -54.8 ± 6.9 | -27 ± 16.2 | -23.1 ± 8.2 |
| **29** | **Cimetidine** | A02BA01 | 51481-61-9 | 0.2 ± 7.2 | -65.4 ± 5.8 | 21.9 ± 3.0 | | -31.2 ± 1.3 | **21.9 ± 3.1** | 1.1 ± 2.5 |
| **30** | **Ciprofloxacin** | J01MA02 | 85721-33-1 | 2.5 ± 1.6 | -2.9 ± 3.2 | 3.3 ± 1.1 | | -6.7 ± 1.9 | **39 ± 3.6** | 6.7 ± 2.6 |
| 31 | Cisplatin | L01XA01 | 15663-27-1 | -4.7 ± 7.9 | -4.1 ± 3.6 | -8.4 ± 4.1 | | -12.6 ± 0.9 | -24.5 ± 12.9 | -1.2 ± 3.1 |
| **32** | **Citalopram** | N06AB04 | 59729-32-7 | -16.7 ± 9.2 | **42.8 ± 4.1** | 8.8 ± 3.3 | | **29.6 ± 3.1** | 8.8 ± 3.3 | -15.6 ± 8.1 |
| 33 | Clarithromycin | J01FA09 | 81103-11-9 | -16.8 ± 2.1 | -14.3 ± 6.2 | -2.4 ± 2.1 | | -13.6 ± 3.3 | -3.9 ± 5.3 | -72.9 ± 1.6 |
| 34 | Clomipramine | N06AA04 | 17321-77-6 | -4.1 ± 0.9 | -12.8 ± 4.8 | -4.5 ± 0.7 | | -12.2 ± 3.9 | 18 ± 3.1 | 14 ± 1.9 |
| **35** | **Clopamide** | C03BA03 | 636-54-4 | 4.5 ± 1.9 | **23.4 ± 2.5** | -14.3 ± 2.1 | | 14.3 ± 2.2 | -11.1 ± 2.3 | 11.6 ± 6.5 |
| 36 | Clopidogrel | B01AC04 | 120202-66-6 | 6.4 ± 4.4 | 1.5 ± 4.1 | 1.9 ± 5.4 | | 0.8 ± 3.1 | -11 ± 8.1 | -8.9 ± 14.9 |
| **37** | **Cyclosporine A** | L04AD01 | 59865-13-3 | -18.1 ± 3.49 | **48.3 ± 2.1** | -5.2 ± 0.4 | | **36.8 ± 2.4** | **36.1 ± 3.1** | **50.2 ± 4.3** |
| 38 | Dapsone | J04BA02 | 80-08-0 | -8.6 ± 4.3 | -27.2 ± 4.2 | -9.8 ± 4.4 | | -24.7 ± 3.1 | 13.4 ± 4.4 | -11.8 ± 3.7 |
| 39 | Desipramine | N06AA01 | 58-28-6 | -10.8 ± 2.3 | 8.3 ± 6.7 | -10.3 ± 2.1 | | 11.6 ± 7.2 | -0.7 ± 3.1 | -37.6 ± 11.8 |
| 40 | Dextromethorphan | R05DA09 | 125-71-3 | 7.3 ± 1.9 | 2.9 ± 3.4 | -0.5 ± 5.8 | | 3.1 ± 0.7 | -3.3 ± 0.3 | 6.2 ± 3.4 |
| **41** | **Diclofenac** | M01AB05 | 15307-86-5 | -15.9 ± 2.5 | -91.4 ± 5 | -15.1 ± 2.1 | | -23.1 ± 2.2 | **26.1 ± 5.3** | -21.2 ± 2.8 |
| **42** | **Diflunisal** | M01AE01 | 22494-42-4 | **26.6 ± 0.9** | -20.5 ± 3.3 | **27.9 ± 1** | | -20 ± 0.5 | -42.4 ± 4.5 | 10 ± 2.1 |
| **43** | **Digitoxin** | C01AA04 | 71-63-6 | -7.2 ± 8.2 | -14.1 ± 4.2 | -7.2 ± 2.7 | | -12.6 ± 2.5 | 4.3 ± 3.3 | **21.5 ± 2.5** |
| 44 | Digoxin | C01AA05 | 244-068-1 | -4.3 ± 7.3 | -14.9 ± 17.8 | -4.8 ± 6.8 | | -12.7 ± 0.8 | -7.4 ± 5.9 | 14.4 ± 4.6 |
| **45** | **Diltiazem** | C08DB01 | 33286-22-5 | -7.3 ± 7.7 | **29.3 ± 2.5** | 8.6 ± 4.3 | | **29 ± 2.6** | 8.5 ± 4.3 | 9.1 ± 7.7 |
| 46 | Doxycycline | J01AA02 | 564-25-0 | 9.7 ± 4.1 | 6.2 ± 0.6 | 9.7 ± 4.1 | | 9.6 ± 1.4 | 3.1 ± 6 | 9.9 ± 3.1 |
| 47 | Duloxetine | N06AX21 | 136434-34-9 | 6.1 ± 7.4 | 12.6 ± 2.6 | 7.6 ± 6.5 | | 11.9 ± 3.1 | 1.9 ± 5.4 | -14.2 ± 4.9 |
| **48** | **Enalapril** | C09AA02 | 75847-73-3 | 9.9 ± 8.3 | -1.5 ± 1.6 | 12.7 ± 4.6 | | -9.5 ± 1.2 | **27.2 ± 2.1** | 5.3 ± 1.8 |
| 49 | Ertapenem | J01DH03 | 202467-69-4 | -28.9 ± 4.8 | -15.8 ± 5.9 | -28.5 ± 4.8 | | -15.1 ± 1.6 | -4.1 ± 6 | 9.9 ± 3.1 |
| 50 | Ethinylestradiol | G03AA01 | 57-63-6 | 7.4 ± 5.9 | -10.5 ± 3.3 | 8.2 ± 5.4 | | -10.1 ± 0.5 | -1.6 ± 3.4 | -35.3 ± 0.8 |
| **51** | **Etorixocib** | M01AH05 | 202409-33-4 | 0.7 ± 3.8 | -20.7 ± 2.9 | -5.4 ± 2.1 | | **31.8 ± 4.3** | -17.2 ± 13.5 | -22.8 ± 2.2 |
| 52 | Felodipine | C08CA02 | 72509-76-3 | -2.2 ± 5.9 | -39.2 ± 1.4 | -9.6 ± 2.8 | | -18.2 ± 1 | -7.4 ± 4.4 | 3.6 ± 2.1 |
| 53 | Fenofibrate | C10AB05 | 49562-28-9 | 16.3 ± 6.6 | 13.1 ± 2 | 10.1 ± 2.9 | | 8.9 ± 0.4 | -4.8 ± 2.1 | 0.2 ± 3.2 |
| 54 | Fludrocortisone | H02AA02 | 127-31-1 | -29.4 ± 8.1 | -24.6 ± 1 | -27 ± 6.7 | | -15.1 ± 0.6 | 0.5 ± 3.9 | -43.9 ± 4.9 |
| **55** | **Fluoxetine** | N06AB03 | 56296-78-7 | **46.5 ± 8.5** | **26 ± 1.5** | -4.4 ± 5.2 | | **25.6 ± 1.2** | -2.4 ± 2 | -18.8 ± 7.8 |
| 56 | Furosemide | C03CA01 | 54-31-9 | 17.5 ± 8.8 | 12.9 ± 2.6 | 18.3 ± 8.3 | | 10.2 ± 3.5 | 4.8 ± 9.4 | -14.4 ± 4 |
| 57 | Glibenclamide | A10BB12 | 93479-97-1 | 13.9 ± 3.4 | 14 ± 9.6 | 12.4 ± 2.8 | | 10.1 ± 3.0 | 16.8 ± 2.5 | 8.1 ± 2.1 |
| 58 | Hydrochlorothiazide | C03AA03 | 58-93-5 | 6 ± 3.9 | 20.7 ± 14.1 | 8.5 ± 4.8 | | 7.9 ± 1.2 | 5.5 ± 2.2 | 9.9 ± 3.5 |
| 59 | Hydrocortisone | H02AB09 | 50-23-7 | 8.5 ± 4.8 | -4.3 ± 2.7 | 13.9 ± 9.6 | | -9.5 ± 2.6 | -0.1 ± 2.8 | -9.9 ± 2.7 |
| 60 | Ibuprofen | M01AE01 | 15687-27-1 | -5.1 ± 3.6 | -33.7 ± 1.4 | -7.5 ± 2.1 | | -26.1 ± 0.6 | -20.2 ± 5.9 | -44.8 ± 7.4 |
| 61 | Imatinib | L01XE01 | 152459-95-5 | -8.2 ± 5.2 | -42.8 ± 0.3 | -24.9 ± 2.1 | | -20.9 ± 1.1 | 11.2 ± 3.4 | 20.7 ± 14.1 |
| 62 | Imidazole | G01AF20 | 1467-16-9 | -6.3 ± 5.9 | -8.5 ± 1.5 | -3.2 ± 2.0 | | -10.8 ± 1.6 | -4.7 ± 2.1 | 20.7 ± 14.1 |
| **63** | **Imipramine** | N06AA02 | 113-52-0 | 0.08 ± 4.8 | **22.6 ± 5.7** | -3.1 ± 1.6 | | 15 ± 2.1 | -4.3 ± 2.1 | 14.1 ± 4.1 |
| **64** | **Ketotifen** | R06AX17 | 34580-14-8 | **23.9 ± 0.8** | 9.9 ± 8.2 | 7 ± 4.2 | | 10.9 ± 2.6 | 6.4 ± 4.4 | 1.4 ± 9.6 |
| **65** | **Lamivudine** | J05AF05 | 134678-17-4 | **27.4 ± 4.6** | -3.9 ± 4.6 | -3.4 ± 0.3 | | -3.1 ± 1.4 | -5.3 ± 4 | **22.9 ± 4.9** |
| 66 | Levamisole | P02CE01 | 16595-80-5 | -18.4 ± 6.9 | -17.2 ± 5.1 | 19.6 ± 11.8 | | -15.9 ± 3.8 | 18.9 ± 12.5 | -34.3 ± 2.4 |
| **67** | **Levodopa** | N04BA01 | 59-92-7 | 8.1 ± 3.1 | **31.4 ± 0.6** | 8.2 ± 3 | | **30.4 ± 0.2** | **28.1 ± 0.9** | -6 ± 0.5 |
| 68 | Lisinopril | C09AA03 | 83915-83-7 | 4.6 ± 3.8 | -12.6 ± 5 | 6.4 ± 3.1 | | -11.4 ± 3.8 | 1.6 ± 1.9 | 15.7 ± 0.8 |
| 69 | Loperamide | A07DA03 | 34552-83-5 | 16.9 ± 2.3 | -3.6 ± 2.1 | 18.9 ± 0.2 | | -2.7 ± 3.9 | 17.6 ± 1.6 | 1.4 ± 2.1 |
| 70 | Losartan | C09CA01 | 114798-26-4 | 13.5 ± 1.7 | -34.8 ± 1.4 | 11 ± 0.5 | | -27.1 ± 2 | 7 ± 3.8 | 12.9 ± 4.1 |
| 71 | Memantine | N06DX01 | 41100-52-1 | -3.2 ± 3.9 | -21.7 ± 5.1 | -3.9 ± 3.4 | | -16.6 ± 0.8 | 14.8 ± 2.6 | -9.6 ± 1.8 |
| 72 | Meropenem | J01DH02 | 96036-03-2 | -19.1 ± 9.3 | -13.4 ± 4.9 | -19.3 ± 9.1 | | -13.6 ± 4.6 | 0.1 ± 3.2 | 12.1 ± 5.1 |
| 73 | Metformin | A10BD25 | 1115-70-4 | -20.7 ± 14.1 | -23.1 ± 1.4 | -20.4 ± 13.7 | | -18.8 ± 1.3 | -8 ± 4.3 | -8.4 ± 3.1 |
| **74** | **Methotrexate** | L04AX03 | 59-05-2 | **22.3 ± 0.8** | -7.1 ± 2.1 | 19.5 ± 1 | | -6.8 ± 0.9 | 5.4 ± 4.3 | 9.9 ± 4.5 |
| **75** | **Methoxamine** | C01CA10 | 61-16-5 | **24.5 ± 10.5** | -23.9 ± 3.7 | 0.7 ± 11.9 | | -23.9 ± 2.1 | 0.7 ± 11.9 | 1.8 ± 0.9 |
| 76 | Metoclopramide | A03FA01 | 364-62-5 | 7.5 ± 2.1 | 2.5 ± 3.5 | -6.3 ± 1.4 | | 1.4 ± 3.2 | -3.2 ± 2.1 | 4.2 ± 9.4 |
| **77** | **Metoprolol** | C07AB02 | 37350-58-6 | 8.1 ± 6.7 | 1.3 ± 3.8 | -21.4 ± 3.7 | | 0.3 ± 2.5 | -15.1 ± 7.1 | **19.8 ± 4.1** |
| 78 | Metronidazole | G01AF01 | 443-48-1 | 7.7 ± 1.9 | 2.6 ± 3.7 | -4.7 ± 2 | | 0.8 ± 2.2 | -3.1 ± 2 | -16.6 ± 0.4 |
| **79** | **Mifepristone** | G03XB01 | 84371-65-3 | **24.6 ± 0.6** | -19.9 ± 4.4 | 8.5 ± 11.1 | | -15 ± 1.6 | 8.1 ± 11.4 | 5.6 ± 3.7 |
| **80** | **Naproxen** | M01AE02 | 22204-53-1 | -3.1 ± 6.6 | **20.3 ± 1.7** | -3.1 ± 6.6 | | 13 ± 0.1 | 6.7 ± 4 | 11.5 ± 2.7 |
| 81 | Nebivolol | C07AB12 | 99200-09-6 | 5.2 ± 3 | 1.3 ± 2.9 | -4.6 ± 1.9 | | 0.3 ± 3.1 | -2.9 ± 1.7 | -6.6 ± 15.4 |
| **82** | **Nefopam** | N02BG06 | 23327-57-3 | -17.2 ± 10.5 | -13.9 ± 5.1 | -8.3 ± 1.6 | | -11.6 ± 3.2 | -3.2 ± 0.4 | **32.6 ± 3.7** |
| **83** | **Nitrendipine** | C08CA08 | 39562-70-4 | -1.5 ± 7.1 | **26.1 ± 8.1** | 3.4 ± 8.7 | | **15.2 ± 4.5** | 3.2 ± 9.5 | **30.4 ± 0.8** |
| **84** | **Norethindrone** | G03AC01 | G03DC02 | **26.9 ± 5** | -19.7 ± 3.3 | 23.5 ± 4 | | -12.8 ± 9.6 | -3.8 ± 1.6 | -3.1 ± 0.5 |
| 85 | Olmesartan | C09CA | 144689-24-7 | 2.8 ± 2.3 | -32.8 ± 1.4 | 3 ± 2.3 | | -13.7 ± 5.1 | -13.2 ± 1.9 | -7.7 ± 3.5 |
| 86 | Ondansetron | A04AA01 | 103639-04-9 | 8.8 ± 7.9 | -5.7 ± 4.1 | -0.5 ± 5.9 | | -8.3 ± 1.6 | -0.4 ± 5.9 | 10.1 ± 2.3 |
| **87** | **Opipramol** | N06AA05 | 909-39-7 | 22.3 ± 1.8 | **29.2 ± 2.7** | -16.8 ± 3.4 | | **29.5 ± 2.7** | -13.4 ± 3.7 | -1 ± 4.5 |
| 88 | Pantoprazole | A02BC02 | 102625-70-7 | 16.7 ± 2.1 | 10.5 ± 0.9 | 15.2 ± 1.8 | | 12.5 ± 0.3 | -4.5 ± 1.7 | -9.9 ± 3.1 |
| 89 | Pravastatin | C10AA03 | 81131-70-6 | 0.2 ± 4.4 | 4.1 ± 2.6 | -0.4 ± 3.8 | | -10.1 ± 1.4 | -18.5 ± 3.3 | -4.4 ± 9.6 |
| **90** | **Prazosin** | C02LE01 | 19237-84-4 | 9.8 ± 4.3 | 9.9 ± 3.1 | **38.2 ± 3.0** | | 9.7 ± 6.1 | **36.9 ± 4.1** | 5.1 ± 2.3 |
| 91 | Prednisolone | S02BA03 | 50-24-8 | 12.5 ± 4.1 | -21.4 ± 1.3 | -11.1 ± 5.8 | | -16.5 ± 2.4 | -3.3 ± 6.9 | 13.4 ± 4.7 |
| **92** | **Prednisone** | S02BA03 | 53-03-2 | 13.9 ± 3.4 | **19.1 ± 0.8** | 12.5 ± 2.7 | | 13.2 ± 2.6 | -33.3 ± 2.3 | -16.9 ± 2.24 |
| 93 | Probenecid | M04AB01 | 57-66-9 | -2.6 ± 3.2 | 0.4 ± 2.1 | -2.9 ± 2.8 | | -1.1 ± 2.2 | -24.9 ± 8.3 | -17.5 ± 1.8 |
| 94 | Promethazine | R06AD02 | 58-33-3 | -1.3 ± 7.1 | 14.4 ± 6 | 4.3 ± 2 | | 14 ± 0.9 | 4.2 ± 2 | -9.2 ± 1.2 |
| **95** | **Pyrimethamine** | P01BD01 | 58-14-0 | 6.9 ± 9.6 | **31.2 ± 2.3** | -25.8 ± 15.5 | | **31.6 ± 2.7** | -37.1 ± 7.2 | **23.4 ± 0.3** |
| 96 | Propranolol | C07AA05 | 13071-11-9 | -0.9 ± 8.1 | -1.1 ± 7.8 | -21.6 ± 0.7 | | -3.5 ± 2.1 | -27.2 ± 3.6 | -36.3 ± 9.5 |
| **97** | **Quinidine** | C01BA01 | 56-54-2 | **29.1 ± 2.8** | 2.7 ± 1 | 4.4 ± 6.2 | | 5.9 ± 1.9 | 4.4 ± 6.2 | -19.6 ± 7.7 |
| 98 | Ramipril | C09AA05 | 87333-19-5 | 0.3 ± 12 | -5.8 ± 2.3 | -0.7 ± 11.5 | | -6.5 ± 2 | 0.1 ± 6.9 | -31.1 ± 6.9 |
| 99 | Ranitidine | A02BA02 | 66357-35-5 | 7.8 ± 1.8 | 2.9 ± 3.9 | -4 ± 0.4 | | 6.8 ± 0.6 | -0.5 ± 5.8 | 6.1 ± 3.4 |
| **100** | **Reserpine** | C02AA02 | 50-55-5 | 4.7 ± 7.9 | 5.2 ± 2.9 | 5 ± 7.5 | | 12.5 ± 1.7 | 9 ± 2.6 | **24.2 ± 0.6** |
| **101** | **Rifampicin *** | J04AB02 | 13292-46-1 | -8.1 ± 11.5 | **(-239.2 ± 19.2)** | -5.6 ± 7.8 | | **(-215.6 ± 11.9)** | -5.5 ± 4.2 | **(-132.7 ± 13.1)** |
| 102 | Simvastatin | C10AA01 | 79902-63-9 | -4.1 ± 0.6 | -10.5 ± 3.3 | -4.6 ± 0.5 | | -6.7 ± 3.6 | -18.1 ± 15 | -4.2 ± 3.5 |
| 103 | Spironolactone | C03DA01 | 52-01-7 | -3.7 ± 5.3 | -4.8 ± 6.6 | -3.9 ± 5.2 | | -11.5 ± 2.8 | 3.2 ± 2.3 | -7.6 ± 2.1 |
| 104 | Sulfanilamide | J01EB06 | 63-74-1 | 20.1 ± 0.9 | 1.8 ± 2.7 | 21.9 ± 1.9 | | -0.6 ± 0.4 | -13.7 ± 1.4 | -48.1 ± 12 |
| **105** | **Sulfamethoxazole** | J01EE01 | 723-46-6 | -8.6 ± 3.6 | 0.9 ± 0.8 | -13.8 ± 2.2 | | -1.5 ± 1.6 | **36 ± 3.5** | -17.1 ± 2.8 |
| **106** | **Tacrolimus** | L04AD02 | 104987-11-3 | **28.5 ± 3.7** | **69.6 ± 4.8** | -10.3 ± 3.6 | | 13.4 ± 1.1 | -14.2 ± 1.2 | -11.8 ± 2.4 |
| **107** | **Ticlopidine** | B01AC05 | 55142-85-3 | 10.4 ± 4.4 | **31.7 ± 10.4** | -4.3 ± 4.6 | | **31.4 ± 10.1** | -11.3 ± 8.3 | -33.5 ± 3.3 |
| 108 | Triamterene | C03DB02 | 396-01-0 | 5.1 ± 8.8 | 16.7 ± 1.6 | 10.7 ± 2.1 | | 14.1 ± 0.5 | 12.6 ± 3.2 | -10.6 ± 0.6 |
| 109 | Trimethoprim | J01EE01 | 738-70-5± | 9.9 ± 4.2 | -16.3 ± 6.7 | -17 ± 1.9 | | -13.8 ± 4.1 | -13.7 ± 2.8 | -68.1 ± 11.3 |
| **110** | **Trimipramine** | N06AA06 | 521-78-8 | 1.3 ± 2.4 | **24.2 ± 4.5** | 1.8 ± 4.5 | | **34.1 ± 3.8** | -26.1 ± 11.5 | -13.4 ± 3.7 |
| **111** | **Trospium Chloride** | G04BD09 | 10405-02-4 | **27.1± 2.1** | -23.8 ± 4.5 | 28.6 ± 1.8 | | -22.5 ± 2.3 | **28.6 ± 0.7** | 3 ± 9.1 |
| 112 | Valproic acid | N03AG01 | 1069-66-5 | 12.6 ± 2.5 | -3.1 ± 6.1 | 10.2 ± 1.4 | | -9.5 ± 5.1 | -7.6 ± 4 | -47.5 ± 6 |
| **113** | **Verapamil** | C08DA01 | 52-53-9 | **29.3 ± 1.1** | **27.46 ± 3.7** | **38.6 ± 3.4** | | **66.8 ± 1.9** | **24.1 ± 2.3** | **44.2 ± 4.4** |
